# Intensifying Electrochemical Activity of Ti_3_C_2_T_x_ MXene via Customized Interlayer Structure and Surface Chemistry

**DOI:** 10.3390/molecules28155776

**Published:** 2023-07-31

**Authors:** Minmin Hu, Lihong Chen, Yunqi Jing, Yuanyuan Zhu, Jun Dai, Alan Meng, Changlong Sun, Jin Jia, Zhenjiang Li

**Affiliations:** 1 School of Materials Science and Engineering, Qingdao University of Science and Technology, Qingdao 266042, China; 2 College of Environment and Safety Engineering, Qingdao University of Science and Technology, Qingdao 266042, China; 3 Key Laboratory of Spin Electron and Nanomaterials of Anhui Higher Education Institutes, Suzhou University, Suzhou 234000, China; 4 State Key Laboratory of Catalysis, Dalian Institute of Chemical Physics, Chinese Academy of Sciences, Dalian 116023, China; 5 College of Electromechanical Engineering, Qingdao University of Science and Technology, Qingdao 266061, China; 6 State Key Laboratory Base of Eco-Chemical Engineering, College of Chemistry and Molecular Engineering, Qingdao University of Science and Technology, Qingdao 266042, China; 7 Institute for Advanced Interdisciplinary Research (iAIR), University of Ji’nan, Ji’nan 250022, China

**Keywords:** MXene, electrochemical activity, supercapacitor, energy storage

## Abstract

MXene, a new intercalation pseudocapacitive electrode material, possesses a high theoretical capacitance for supercapacitor application. However, limited accessible interlayer space and active sites are major challenges to achieve this high capacitance in practical application. In order to stimulate the electrochemical activity of MXene to a greater extent, herein, a method of hydrothermal treatment in NaOH solution with reducing reagent-citric acid is first proposed. After this treatment, the gravimetric capacitance of MXene exhibits a significant enhancement, about 250% of the original value, reaching 543 F g^−1^ at 2 mV s^−1^. This improved electrochemical performance is attributed to the tailoring of an interlayer structure and surface chemistry state. An expanded and homogenized interlayer space is created, which provides enough space for electrolyte ions storage. The –F terminations are replaced with O-containing groups, which enhances the hydrophilicity, facilitating the electrolyte’s accessibility to MXene’s surface, and makes MXene show stronger adsorption for electrolyte ion-H^+^, providing sufficient electrochemical active sites. The change in terminations further leads to the increase in Ti valence, which becomes more prone to reduction. This work establishes full knowledge of the rational MXene design for electrochemical energy storage applications.

## 1. Introduction

Electrochemical energy storage is the key technology determining the future of transportation, mobile communication, and renewable energy utilization [1,2,3], which motivates the development of devices that combine high energy density and power density while being safe to use [4]. A supercapacitor is unique as an energy storage device owing to its high power density, stable and longer operating lifespan, superior safety tolerance, and fast charging and discharging rate [5,6]. In particular, pseudocapacitors can store more charge than electrical double-layer (EDL) capacitors and have a faster charging/discharging rate compared to batteries [4,7]. This makes them attractive but requires an excellent active electrode material. MXene, a large family of two-dimensional (2D) transition-metal carbides and/or nitrides, has been demonstrated as a promising intercalation pseudocapacitive material due to its metallic conductivity, surface hydrophilicity, ionic channels between 2D sheets, and redox-active surface [4,8,9,10,11].

MXene is expressed as the formula of M*_n_*_+1_X*_n_*T_x_ (*n* = 1, 2 or 3), where M represents transition metal(s), X is carbon and/or nitrogen, and T_x_ stands for the surface functional groups, such as –O, –OH, and –F [12,13,14]. In acidic aqueous electrolytes, Ti_3_C_2_T_x_, the representative member and the most widely used MXene, has a high theoretical capacitance, which is attributed to the efficient EDL charge accumulation utilizing the interlayer space and the fast pseudocapacitive proton intercalation/deintercalation with surface redox reaction [15]. However, limited accessible interlayer space and active sites are major challenges for MXene to achieve high capacitance in practical applications. Therefore, tremendous efforts have been made to overcome the restacking of MXene layers and conduct surface chemistry state regulation in recent years.

First of all, to create a large interlayer space that can accommodate more electrolyte ions and intensify fast ion diffusion into electrochemically active sites deep inside the interlayers, researchers adopted various methods, including intercalation of foreign species [16,17,18,19,20], ultrasonication [21], and doping [22,23], to expand the interlayer voids. In particular, the intercalation of foreign metal cations has been demonstrated to be an effective method to improve the electrochemical performance of Ti_3_C_2_T_x_ MXene, which can not only enlarge the interlayer voids but also homogenize the interlayer spacing. For example, an extraordinarily high intercalation capacitance of 415 F cm^−3^ at 5 A g^−1^ of Ti_3_C_2_T_x_ was obtained in a H_2_SO_4_ electrolyte after intercalation treatment using KOH and KOAc [20]. Moreover, monovalent Li^+^, Na^+^, or Cs^+^ ions also exhibit a superior intercalation effect, promoting the intercalation of electrolyte ion H^+^ [16].

Except for the interlayer structure, the surface functional groups (T), which are in direct contact with the electrolyte, greatly influence the capacitance of MXene electrodes [9,24,25]. EDL capacitances of Ti_3_C_2_T_x_ MXene depend on its terminated elements, where the termination atom with a lower electronegativity facilitates the electrostatic accumulation of electrons at the electrode surface [24]. During the pseudocapacitive electrochemical process, bonding between oxygen functional groups rather than fluorine terminations and the hydrogen ion from an acidic electrolyte accompanied with the partial electron transferring with corresponding modification of the Ti atom oxidation state of the Ti_3_C_2_T_x_ surface layers always occurs [26,27]. Obviously, among the terminations of MXene, O-containing groups can not only increase the EDL capacitance due to their lower electronegativity but could also improve the pseudocapacitance working as active sites for proton bonding/debonding [13,24,26]. However, in general, the pristine MXene obtained by removing the “A” elements of its corresponding MAX phase (ternary carbides and nitrides, where M is an early transition metal, A is an A-group element, and X is carbon and/or nitrogen) by hydrofluoric acid or LiF-HCl etching is always terminated with a mass of –F groups. Therefore, significant advances have been made in MXene surface modification over the years. Alkali treatment is a common and convenient method to remove the surface F terminations, but the defluorination effect of a single alkaline aqueous solution is not obvious [19,20]. Annealing can further remove the surface functional groups; however, unfortunately, MXene materials are easily oxidized during the annealing process even with inert gas protection, and the oxidation products like TiO_2_ with unsatisfactory conductivity would affect the electrochemical behavior [28]. Therefore, a more efficient method for tuning the terminal groups on MXene is highly desired.

In addition, the initial oxidation state of the transition metal is crucial for the surface redox between H^+^ and the MXene [29]. Wang et al. demonstrated that the partial oxidation of MXene can intensify pseudocapacitive Li^+^ intercalation into Ti_3_C_2_T_x_ MXene from neutral water-in-salt electrolytes due to the higher oxidation state of Ti at the open circuit potential, which is more prone to reduction [4]. Tang et al. also tuned the electrochemical performance of Ti_3_C_2_T_x_ MXene by controlling the transition metal stoichiometry employing in situ anodic oxidation [30]. Therefore, the chemical states of transition metals should be taken into account in the regulatory modification of MXene.

**Figure 1:**
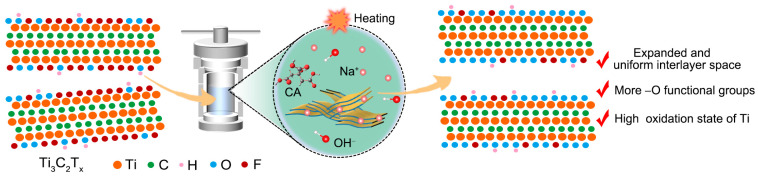
Schematic of the modification strategy for MXene. Hydrothermal treatment method in NaOH solution with the reducing reagent CA is employed, which endows the MXene with expanded and uniform interlayer space, sufficient –O functional groups, and high oxidation state of Ti.

Inspired by the above motivations, we developed a simple and effective hydrothermal strategy to modify the interlayer structure and surface chemistry state of MXene as shown in Figure 1, where Ti_3_C_2_T_x_ MXene sheets were heated in the aqueous solution containing NaOH and reducing reagent citric acid (CA) in a Teflon-lined stainless steel autoclave at 180 °C for 24 h. Surprisingly, this method not only leads to the expansion and homogenization of interlayer space but also shows high efficiency in regulating surface chemistry state, replacing –F with –O functional groups, and increasing the oxidation state of Ti. Importantly, the MXene is not oxidized due to the presence of reducing reagent. The enlarged interlayer space conducive to ion accommodation, the enhancement of –O terminations providing better hydrophilicity and abundant electrochemically active sites, and higher oxidation state of transition metal that is easily reduced intensifies electrolyte ions intercalation, resulting in the emergence of higher current densities and a pair of clear redox peaks in cyclic voltammogram (CV) curves. Consequently, a record high capacitance of 543 F g^−1^ at 2 mV s^−1^ was obtained. This work not only provides desired strategies for effectively enhancing the electrochemical performances of MXenes and yields valuable insights in comprehensive understanding their charge storage mechanisms, but also establishes full knowledge of the rational MXene design for practical electrochemical energy storage application.

## 2. Results and Discussion

### 2.1. Material Characterization

**Figure 2:**
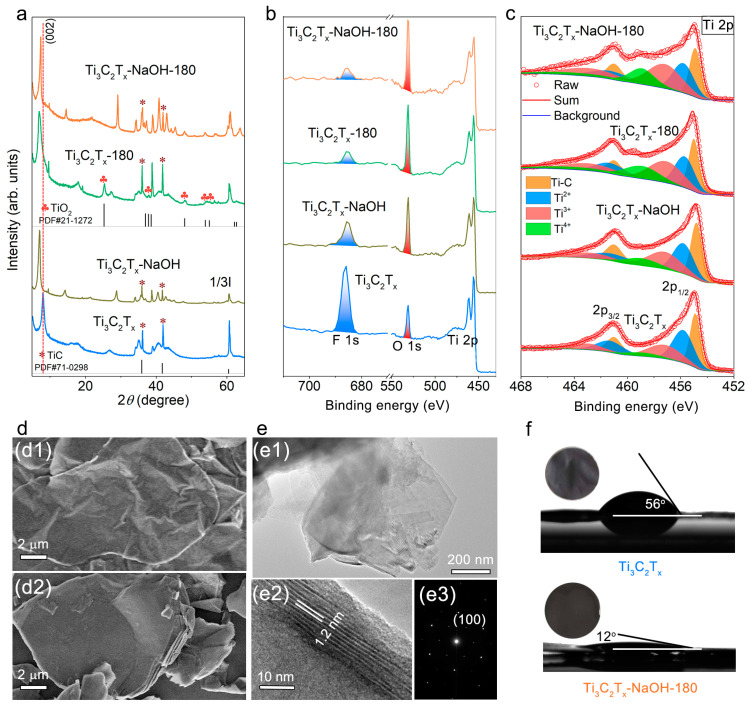
(**a**) XRD patterns, (**b**) XPS spectra, and (**c**) component peak-fitting of Ti 2p spectra for pristine Ti_3_C_2_T_x_ MXene, Ti_3_C_2_T_x_-NaOH, Ti_3_C_2_T_x_-180, and Ti_3_C_2_T_x_-NaOH-180; (**d**) SEM images of (**d1**) pristine Ti_3_C_2_T_x_ MXene and (**d2**) Ti_3_C_2_T_x_-NaOH-180; (**e**) (**e1**,**e2**) TEM images and (**e3**) SAED pattern of Ti_3_C_2_T_x_-NaOH-180; (**f**) Contact angle of water on the pristine Ti_3_C_2_T_x_ MXene film and Ti_3_C_2_T_x_-NaOH-180 MXene film (inserts show the optical photographs of corresponding films).

As shown in Figure 2a, after the treatment by NaOH at room temperature, the (002) peak of the obtained Ti_3_C_2_T_x_-NaOH MXene sample became sharper and shifted to a lower 2*θ* angle in the X-ray diffraction (XRD) pattern, indicating that the spontaneous intercalation of Na^+^ ions between layers induces a homogeneity and expansion in space between single Ti_3_C_2_ sheets, which undoubtedly provides enough space for electrolyte ion storage. Meanwhile, –F terminal groups were replaced with oxygen-containing functional groups after basic solution treatment, noticeable by the quick decrease in the intensity of F1s peak and the increase in the intensity of O1s peak in X-ray photoelectron spectroscopy (XPS) spectrum of Ti_3_C_2_T_x_-NaOH in comparison to that of Ti_3_C_2_T_x_ as shown in Figure 2b. More –O functional groups on the MXene surface can provide more electrochemical active sites, intensifying pseudocapacitive H^+^ intercalation [26]. Here, we note that the –Cl group was also observed in the pristine Ti_3_C_2_T_x_ MXene. But, its content is negligible and has no obvious change after NaOH treatment (Figure S1). Therefore, here, we did not pay further attention to the study of the effect of –Cl terminations. In addition, from the high-resolution of Ti 2p spectra (Figure 2c), we observe that the peaks of Ti 2p_3/2_ and Ti 2p_1/2_ can be differentiated with four spin-orbit doublet characteristics of Ti, Ti^2+^, Ti^3+^, and Ti^4+^ [31]. Interestingly, the area ratio of the Ti^3+^ to Ti^2+^ curves in Ti_3_C_2_T_x_-NaOH is higher than that in pristine Ti_3_C_2_T_x_, which implies that the modification of surface functional groups also leads to the increase in the valence state of Ti. The high valence state Ti^3+^ is easier to be reduced, contributing significantly to the redox reaction activity of MXene during the charge–discharge process.

Ti_3_C_2_T_x_-180, obtained by hydrothermal treatment as described in the Experiment section, was investigated to shed light on the effect of heating on MXene. As shown in Figure 2a, hydrothermal heating also leads to the expansion of interlayer space, which is confirmed by the downshift to a lower angle of the (002) peak. But, the full width at half maximum width (FWHM) of the (002) peak does not show a decrease as in Ti_3_C_2_T_x_-NaOH, indirectly implying that the intercalation of cations can increase the homogeneity of the layer domains that now have identical interlayer spacings [32]. In addition, after hydrothermal treatment, Ti_3_C_2_T_x_ MXene is partially oxidized to TiO_2_ (Figure 2a). Fortunately, hydrothermal heating also can cause an increase in the content of O-containing functional groups and the valence of Ti (Figure 2b,c).

To take advantage of the alkaline and hydrothermal effect on the tailoring of structure and chemical state, the MXene was heated in the NaOH solution, which generates the sample denoted as Ti_3_C_2_T_x_-NaoH-180. Compared with pristine Ti_3_C_2_T_x_, the obtained Ti_3_C_2_T_x_-NaoH-180 sample still maintains the lamellar morphology confirmed by their scanning electron microscopy (SEM) images (Figure 2d1,d2). Also, it is very thin and disperses as an individual layer or stack of several layers confirmed by transmission electron microscopy (TEM) images (Figure 2(e1) and Figure S2a), exhibiting a hexagonal structure demonstrated by its selected area electron diffraction (SAED) pattern (Figure 2(e3)) and high-resolution TEM image (Figure S2b). Moreover, it shows an expanded and uniform interlayer distance (*d* = 1.2 nm) due to the insertion of Na^+^ ions along with the heating effect (Figure 2a,(e2)) and there is no formation of TiO_2_ owing to the addition of reducing agent CA as shown in Figure 2a. Importantly, most –F terminal groups were substituted by O-containing groups, and the valence of Ti increases dramatically (Figure 2b,c). The enhancement of the content of O-containing groups would increase the hydrophilicity of MXene, which can be demonstrated by the considerable decrease in the contact angle of water on the Ti_3_C_2_T_x_-NaOH-180 MXene film (Figure 2f). This is beneficial for the electrolyte’s accessibility to the film’s surface and accelerates ion diffusion in energy storage. In addition, all fabricated samples contain impurity phase TiC, as shown in Figure 2a, which inherit from the precursor Ti_3_AlC_2_. TiC has no electrochemical activity in H_2_SO_4_ solution; therefore, they would not contribute to the electrochemical performance.

### 2.2. Density Functional Theory Calculations

**Figure 3:**
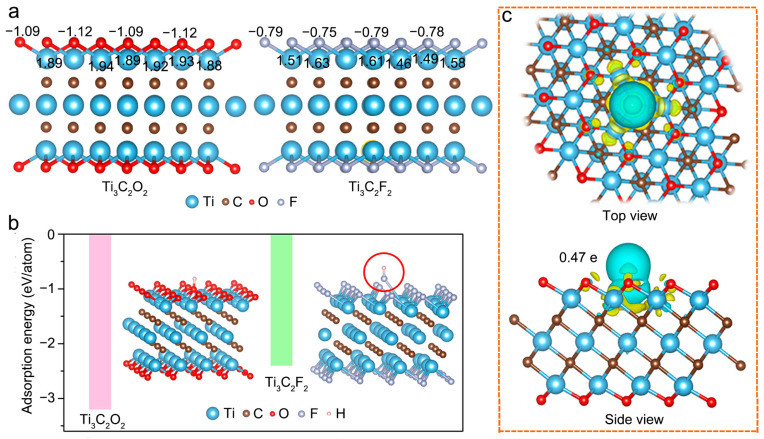
(**a**) Distribution of element valence in Ti_3_C_2_O_2_ and Ti_3_C_2_F_2_; (**b**) Adsorption energies of H atoms over Ti_3_C_2_O_2_ and Ti_3_C_2_F_2_ (inserts show the schematic adsorption configuration of single H atom on top of O or F atoms of Ti_3_C_2_O_2_ or Ti_3_C_2_F_2_); (**c**) Top view and side view of charge density difference during the adsorption process of single H atom on the surface of Ti_3_C_2_O_2_, in which yellow and green regions represent the electron accumulation and depletion area, respectively.

Here, we note that the role of surface chemistry on H^+^ charge storage behavior was always indirectly proved by experimental results in previous reports [20,26,28] without sufficient direct evidence on verifying their relationship. Computational simulations are effective tools to complement experiments for unveiling the effect of terminated MXene on electrochemical performance [24,33]. First, we focused on the structural properties of Ti_3_C_2_T*_x_* MXene terminated with different functional groups, i.e., Ti_3_C_2_O_2_ and Ti_3_C_2_F_2_. As shown in Figure 3a, the structures of Ti_3_C_2_O_2_ and Ti_3_C_2_F_2_ were hexagonal and stacked in a sequence of O/F–Ti–C–Ti–C–Ti–O/F, forming seven-layer structures with O or F functional groups occupying face-centered cubic sites [34]. Based on the Bader charge analysis (Figure S3, Tables S1 and S2), we observed that more electrons transfer from Ti atoms to O atoms compared to those transferring to F atoms, and consequently, Ti atoms in Ti_3_C_2_O_2_ lose more electrons with higher valence (Figure 3a). This result is consistent with Ti 2p XPS spectra in Figure 2c, in which the area ratio of the Ti^3+^ to Ti^2+^ curves in these processed samples is higher than that in pristine Ti_3_C_2_T_x_ due to more –O functional groups being terminated. The higher the oxidation valence of Ti, the more prone it is to be reduced when the electrode is discharged, i.e., during the H^+^ insertion process, which contributes considerably to the redox reaction activity, intensifying the pseudocapacitive H^+^ intercalation [4].

In addition, a suitable electrode material for rechargeable supercapacitors should possess a relatively strong ability for adsorbing atoms [33,34,35]. Therefore, we then examined the adsorption behaviors of a single H atom on the surface of Ti_3_C_2_O_2_ and Ti_3_C_2_F_2_. In general, negative adsorption energy manifests that the adsorption process can occur spontaneously while positive adsorption energy means that the occurrence of this adsorption process needs an additional energy input [34]. As shown in Figure 3b, all the adsorption energies of single H atoms over Ti_3_C_2_O_2_ and Ti_3_C_2_F_2_ were negative with values of −3.2 and −2.4 eV/atom, respectively, indicating that all these adsorption processes are thermodynamically favorable. Notably, the adsorption energy of a single H atom over Ti_3_C_2_O_2_ is lower than that of Ti_3_C_2_F_2_, suggesting H can be more favorably stabilized on the Ti_3_C_2_O_2_ monolayer as compared to the Ti_3_C_2_F_2_ monolayer. More importantly, the F bonded with H repulses with Ti, causing the instability of the whole structure, as shown in the structure circled in red in Figure 3b. However, for Ti_3_C_2_O_2_, the H atom can easily absorb on Ti_3_C_2_O_2_ and the adsorbed H transfers 0.47 e to the surrounding atoms, suggesting a strong bonding between H and the Ti_3_C_2_O_2_ substrate (Figure 3c). These above results elucidate that the –O groups with more negative charges can endow strong adsorption sites for capturing electrolyte ion H^+^, and the Ti in Ti_3_C_2_O_2_ can accumulate electrons accompanied by the reduction in Ti atoms upon discharging to achieve a more stable state of the system. In other words, the capacitance of Ti_3_C_2_O_2_ originates from the redox reaction. For Ti_3_C_2_F_2_, it is a detriment to participate in pseudocapacitive energy storage processes due to its unstable structure. This analysis agrees with the experimental results that the protonation of O functional groups accompanied by the change in Ti oxidation state occurs during the electrochemical process [27].

### 2.3. Electrochemical Performances of Electrodes

**Figure 4:**
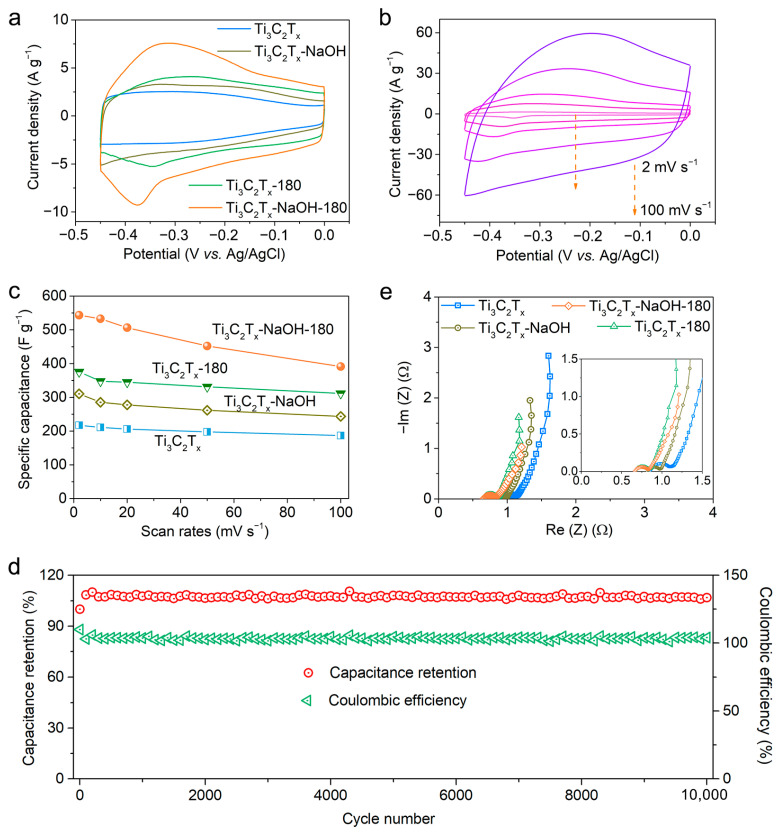
Electrochemical performance of pristine Ti_3_C_2_T_x_ MXene, Ti_3_C_2_T_x_-NaOH, Ti_3_C_2_T_x_-180, and Ti_3_C_2_T_x_-NaOH-180. (**a**) CV curves at a scan rate of 10 mV s^−1^; (**b**) CV curves at various scan rates for Ti_3_C_2_T_x_-NaOH-180 (the dashed line with an arrow represents a change in scan rates from 2 to 100 mV s^−1^); (**c**) specific capacitances as a function of scan rate calculated from the CV curves; (**d**) Nyquist plots recorded from 1 Hz to 100 kHz at open-circuit voltage with an amplitude of 5 mV; (**e**) cycling stability and Coulombic efficiency of Ti_3_C_2_T_x_-NaOH-180 for 10,000 cycles.

The electrochemical performance of the Ti_3_C_2_T_x_-NaOH-180 electrode and its counterparts were estimated in the standard three-electrode systems, as presented in Figure 4. Figure 4a shows the CV curves of the Ti_3_C_2_T_x_ electrodes in 1 mol L^−1^ H_2_SO_4_ electrolyte at a scan rate of 10 mV s^−1^. A higher current density is observed for Ti_3_C_2_T_x_-NaOH, Ti_3_C_2_T_x_-180, and Ti_3_C_2_T_x_-NaOH-180 compared to pristine Ti_3_C_2_T_x_, indicative of the positive role that these treatments play in the electrochemical performance of MXene. Here, we note that Ti_3_C_2_T_x_-180 exhibits a higher current density than that of Ti_3_C_2_T_x_-NaOH, which results from the fact that Ti_3_C_2_T_x_-180 possesses more O-containing terminal groups and a higher oxidation state of Ti, even though there is an uneven interlayer space and the formation of a small quantity of TiO_2_ in Ti_3_C_2_T_x_-180 MXene. This also indicates that surface chemical composition and state, instead of the interlayer structure, play a more important role in the regulation of electrochemical performance for MXene. Notably, the highest current density and a pair of clearer and broadened oxidation−reduction peaks emerging from about −0.2 V to the most negative potential are observed for the Ti_3_C_2_T_x_-NaOH-180 electrode. The CV plots of the Ti_3_C_2_T_x_-NaOH-180 and other prepared electrode materials at various scan rates ranging from 2 to 100 mV s^−1^ are presented in Figure 4b and Figure S4, respectively. In accordance with these CV curves, the profiles of scan rate versus capacitance are shown in Figure 4c. A maximum specific capacitance of 543 F g^−1^ at 2 mV s^−1^ is accomplished by the Ti_3_C_2_T_x_-NaOH-180 electrode, increasing by about 150% compared to that of pristine MXene and outstripping the reported capacitance of most Ti_3_C_2_T_x_-based electrode materials (Table S3). Moreover, it still exhibits a favorable capacitance of 391 F g^−1^ at a high scan rate of 100 mV s^−1^. Importantly, the Ti_3_C_2_T_x_-NaOH-180 electrode manifests excellent permanency throughout the cycles, with more than 100% capacitance retention and nearly 100% Coulombic efficiency over 10,000 cycles (Figure 4d).

In addition, we specifically investigated the electrochemical impedance spectroscopy (EIS) of these processed Ti_3_C_2_T_x_ MXene electrodes (Figure 4e). The equivalent circuit simulation results (Figure S5 and Table S4) show that the equivalent series resistance (ESR) determined from the intercept of the -*x*-axis, and the charge transfer resistance (*R*_ct_) determined from the diameter of the semicircle in the high-frequency range [36], for the processed MXene electrodes, are slightly smaller than that of pristine species, indicating that the electrode conductivity is not significantly affected after alkaline or hydrothermal treatment. Importantly, in terms of the Ti_3_C_2_T_x_-NaOH-180 electrode, its ESR and *R*_ct_ are the smallest among them, which accounts for its better electrochemical performance.

### 2.4. Kinetic and In Situ Electrochemical EIS Analysis

**Figure 5:**
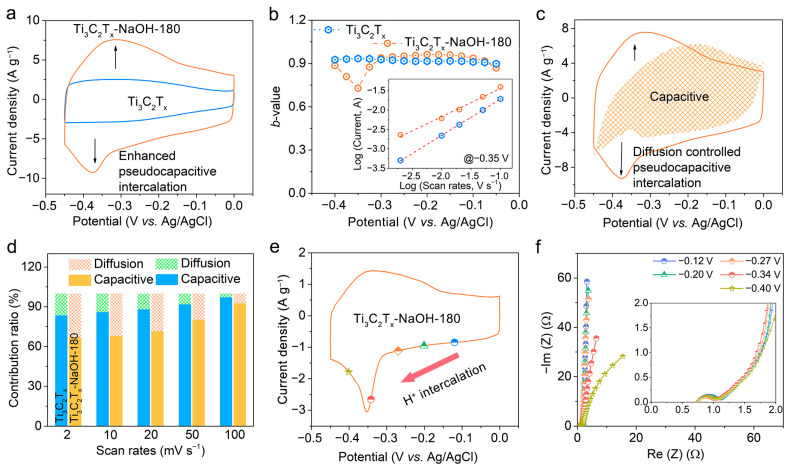
(**a**) CV curves of pristine Ti_3_C_2_T_x_ and Ti_3_C_2_T_x_-NaOH-180 electrodes in H_2_SO_4_ electrolytes at a scan rate of 10 mV s^−1^; (**b**) *b* values for the Ti_3_C_2_T_x_ and Ti_3_C_2_T_x_-NaOH-180 electrodes plotted as a function of potential vs. Ag/AgCl. Inset: power-law dependence of current on sweep rate at –0.35 V; (**c**) CV profile collected at 10 mV s^−1^ with hatched portions showing the contribution of the capacitive-limited process; (**d**) Capacitive contribution ratio for the Ti_3_C_2_T_x_ and Ti_3_C_2_T_x_-NaOH-180 electrodes at different scan rates ranging from 2 to 100 mV s^−1^; (**e**,**f**) In situ EIS spectra (**f**) of the Ti_3_C_2_T_x_-NaOH-180 electrode collected at different potentials vs. Ag/AgCl from 0.01 Hz to 100 kHz with an amplitude of 5 mV on the intercalation branch of CV profile (**e**).

For the Ti_3_C_2_T_x_-NaOH-180 electrode, we note that a pair of clearer and broadened peaks emerge in the CV curves, which are located at similar potentials from about −0.2 V to the most negative potential on the cathodic and anodic branches (Figure 5a). To shed light on the charge storage behavior during this charge−discharge process, we performed a series of tests of the electrode at the specific charged and discharged states (Figure 5b–f). Figure 5b illustrates the *b* value in kinetic analysis at various potentials corresponding to the discharge branch of the CV curves, which can indicate the dominating charge storage mechanism of the process. The *b* values were obtained by plotting the logarithm of the current (log *i*) at a certain potential on the discharge branch of the CV curves as a function of the logarithm of the scan rate (log *ν*) and estimating the slope of the curve of log *i* = log *a* + *b* log *ν* (Figures S6 and S7) [37]. Obviously, the *b* values are close to 1 for the pristine Ti_3_C_2_T_x_ electrode over the entire voltage range, which means that the electrochemical process is dominated by surface-controlled charge storage behavior.

However, in terms of the Ti_3_C_2_T_x_-NaOH-180 electrode, the *b* value at −0.05 V ~ −0.3 V is close to 1, while there is a valley in the *b* value curve at −0.3 V ~ −0.4 V corresponding to the peak in the discharge curve. This indicates that the electrochemical processes corresponding to the peaks in CV plots are dominated by diffusion-controlled charge storage behavior. In addition, from the calculation results of the capacitive and diffusion-limited contributions to the total capacitances, we can see that there is a noticeable and prevailing contribution of diffusion-limited processes to the total capacitance at the peaks in CV curves (Figure 5c and Figure S8). Therefore, the diffusion-limited contributions to the total capacitances for Ti_3_C_2_T_x_-NaOH-180 are larger than that of pristine MXene (Figure 5d). In Ti_3_C_2_T_x_ MXene, there are two kinds of charge storage behavior: EDL charge accumulation utilizing the interlayer space and the pseudocapacitive protonation of the O-terminated surface. Therefore, the electrochemical processes should be dominated by EDL charge storage behavior due to its capacitive feature in pristine MXene, while the electrochemical processes are divided into two parts in Ti_3_C_2_T_x_-NaOH-180 MXene: the rectangular-shaped CV curve with capacitive feature (the *b* value of close to 1) should result from the EDL charge storage process, and the peaks in CV curve with diffusion-limited behavior feature (the *b* value of lower than 1) should derive from pseudocapacitive charge storage process.

We can also understand the electrochemical process of Ti_3_C_2_T_x_-NaOH-180 MXene from the in situ electrochemical EIS results. Figure 5f shows the Nyquist plots collected at different applied reducing potentials that are in good agreement with the CV data (Figure 5e). In the low-frequency range, the imaginary part value of impedance, −Im (Z), is expected to be linked to the gravimetric differential capacitance, C (−Im (Z) = 1/ωC), where ω is the angular frequency of the alternating current, and C should thus be comparable to that obtained from the related CV curves [36,38]. As the reactions proceed, i.e., H^+^ intercalation, the capacitance C is increasing. Therefore, the value of −Im (Z) is decreasing due to the inverse relationship between them, as discussed above. Notably, from −0.12 V to −0.27 V, the value of −Im (Z) decreases slightly, whereas from −0.27 V to −0.34 V, it decreases dramatically, which should result from more pseudocapacitive charge accumulation at the peak potentials. In the low-frequency range of Nyquist plots, as the intercalation proceeds, the plot remains vertical at all times, represented by a CPE with a fractional exponent *α* ≈ 0.86 (Table S5), which implies that there is a surface-controlled EDL charge storage process. But, when the electrode is discharged to peak potential, the plot becomes more and more inclined, represented by a CPE with a fractional exponent *α* ≈ 0.76 (Table S5), elucidating that there is a diffusion-controlled pseudocapacitive charge storage process. This is highly consistent with the kinetic analysis result.

### 2.5. Ex Situ XRD Analysis

**Figure 6:**
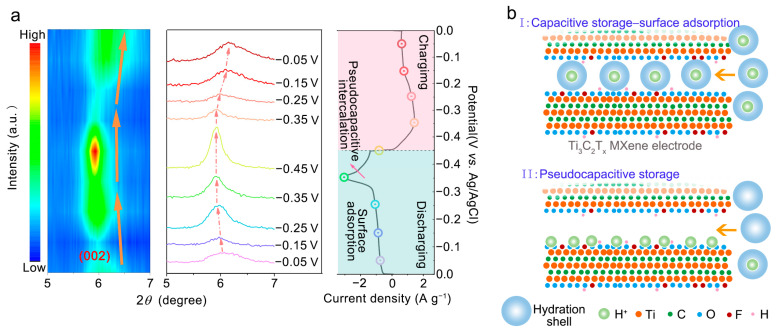
(**a**) Ex situ XRD tests of the Ti_3_C_2_T_x_-NaOH-180 electrode during various electrochemical steps (arrows represent the change direction of 2*θ* (left) and circles represent the selected charge/discharge voltage (right)); (**b**) schematic pictures of the capacitive and pseudocapacitive conditions formed inside the MXene electrodes.

To facilitate a deeper understanding of the specific electrochemical behavior of Ti_3_C_2_T_x_-NaOH-180, ex situ XRD measurements were conducted at the specific charged and discharged states marked on the CV curves to monitor the structural evolution during the charging–discharging process. Figure 6a displays ex situ XRD patterns with 2*θ* ranging from 5° to 7° operating at room temperature. Compared to the previous report on cation intercalation into Ti_3_C_2_T_x_, which showed reversible and continuous changes in the interlayer space, the mechanism of H^+^ intercalation here appears to be more complex. There was both expansion or shrinkage and invariability of the *c*-lattice parameter (interlayer space) during a single charge or discharge. Specifically, during the discharging process, the *c*-lattice parameter continuously enlarged from −0.05 V to −0.35 V vs. Ag/AgCl, which was confirmed by the shift in the (002) peak to a lower angle, and then it remained nearly constant from −0.35 V to −0.45 V vs. Ag/AgCl, as evidenced by the immobility of the position of the (002) peak. During the charging process, the *c*-lattice parameter remained unchanged from −0.45 V to −0.35 V vs. Ag/AgCl, and then it shrank continuously from −0.35 V to −0.05 V vs. Ag/AgCl. The evolution of the voltage-dependent *c*-lattice parameter is nearly reversible. Notably, a striking feature was observed where the expansion/shrinkage of the *c*-lattice parameter occurred at potentials corresponding to rectangular-shaped CV plots, while the invariability of the *c*-lattice parameter was observed at redox peak potentials.

This implies that there are different charge storage behaviors in the whole electrochemical process, as discussed below. It has been revealed that the electrochemical behavior of MXene depends on the state of intercalated ions [39,40]. When ions are fully hydrated, an EDL can form without charge transfer between the ions and electrodes. However, once the ions are partially dehydrated and directly adsorbed onto the MXene surface, orbital hybridization occurs with a surface termination species, which leads to the depletion of the electrostatic potential difference due to charge redistribution, rendering the pseudocapacitance dominant [39,40]. This can help explain the aforementioned distinction in electrochemical behavior at different potentials for the Ti_3_C_2_T_x_-NaOH-180 electrode. The interlayer distance of pristine MXene is much larger than the H^+^ ion diameter, which can accommodate the inserted H^+^ ions without any structure change. In our work, however, the *c*-lattice parameter expanded continuously when the electrode was discharged from −0.05 V to −0.35 V vs. Ag/AgCl. Therefore, the H^+^ should have been intercalated in a hydrated state, leading to the enlargement of interlayer space owing to their larger radius. However, the hydration shell would prevent orbital coupling between MXene and the intercalated H^+^ ions, leading to the formation of an electric-double layer and capacitive behavior, as shown in Figure 6b (top). Therefore, the CV curve in this range of potentials exhibits a rectangular shape, which agrees with the result of the surface-controlled electrochemical process in kinetic analysis and the vertical plot of the low-frequency region in EIS spectra.

The *c*-lattice parameter remained nearly constant at potentials from −0.35 V to −0.45 V vs. Ag/AgCl, indicating that partial or full desolvation occurs at the MXene–electrolyte interface and that the H^+^ ion is inserted in a dehydrated state rather than a hydrated state. Once the cations are partially or fully dehydrated and adsorb onto the MXene surface, charge transfer occurs due to orbital coupling of the cation states with the MXene states, particularly for oxygen surface-termination groups, resulting in pseudocapacitive behavior [39,40], as shown in Figure 6b (bottom). Consequently, the CV curve in this potential range exhibits a significant reduction peak, which is consistent with the result of the diffusion-controlled electrochemical process in kinetic analysis and the inclined plot of the low-frequency region in EIS spectra.

All experimental evidence suggests that the cathodic and anodic peaks observed in CV curves for Ti_3_C_2_T_x_-NaOH-180 in H_2_SO_4_ electrolytes originate from a diffusion-limited Faradaic process involving the protonation of oxygen functional groups. In Ti_3_C_2_T_x_-NaOH-180, the content of −O terminations is higher, providing better hydrophilicity and abundant electrochemical active sites. The oxidation state of the transition metal also becomes higher and easier to reduce, which intensifies a stronger unhydrated H^+^ pseudocapacitive intercalation, resulting in the emergence of a clear pair of redox peaks in the CV curve. Additionally, the interlayer space is expanded and homogenized by Na^+^ intercalation and the hydrothermal effect, which provides enough space for EDL capacitive ion storage. This results in the emergence of higher current densities at potentials without peaks in the CV curve.

## 3. Experimental Section

### 3.1. Synthesis of Ti_3_C_2_T_x_ MXene

Ti_3_C_2_T_x_ MXene was synthesized using a mild method, which involved the selective etching of Al from Ti_3_AlC_2_ using in situ hydrofluoric acid (HF)-forming etchant, as previously reported [41]. The etching solution was prepared by adding 1.6 g lithium fluoride (LiF) to 20 mL hydrochloric acid (HCl, 9 mol L^−1^) and then stirring for 5 min. Next, 1 g Ti_3_AlC_2_ powder was slowly added to the etchant at room temperature until no bubbles were detected in the reaction system. The etching solution was decanted, and the solids were filtered. A dilute HCl solution was then added to remove the residual LiF. Fresh deionized (DI) water was added to wash the obtained powder several times until the pH value of the liquid reached 5. Finally, the washed powder was vacuum-dried at 60 °C.

### 3.2. Modification of Interlayer Structure and Surface Chemical State

The Ti_3_C_2_T_x_ powder was immersed in 1 mol L^−1^ NaOH solution and ultrasonicated for 40 min. The mixture was then decanted and filtered, and DI water was added to wash the powders several times. The resulting Ti_3_C_2_T_x_-NaOH powder was dried at 60 °C. The Ti_3_C_2_T_x_-180 powder was obtained by treating Ti_3_C_2_T_x_ powder in a Teflon-lined stainless steel autoclave under hydrothermal conditions at 180 °C for 24 h. For Ti_3_C_2_T_x_-NaOH-180, 1.2 g CA was dissolved in 60 mL NaOH solution under magnetic stirring. Then, 0.2 g Ti_3_C_2_T_x_ MXene powder was added to the solution, followed by ultrasonication for 40 min. Afterward, the mixture was transferred into a 100 mL Teflon-lined stainless steel autoclave at 180 °C for 24 h. After natural cooling, the resulting powder was washed with DI water several times and dried at 60 °C.

### 3.3. Material Characterizations

XRD patterns of the samples were recorded using a Rigaku Ultima IV X-ray diffractometer with Cu Kα radiation (λ = 1.5418 Å). XPS analysis was performed on a Thermo Scientific ESCALAB XI X-ray photoelectron spectrometer with an Al-Kα excitation source to characterize the chemical compositions. The microstructure morphology was characterized using a TESCAN MIRA LMS field emission SEM, and more detailed structural information was acquired using a JEM-2100PLUS TEM. The hydrophilicity of the samples was studied using a contact angle meter (LSA 100, LAUDA Instruments GmbH, Lauda-Königshofen, Germany).

### 3.4. Electrochemical Measurements

To evaluate the electrochemical performance of the MXene materials, a three-electrode configuration was used with the MXene-based electrode as the working electrode, platinum as counter electrode, and Ag/AgCl in saturated KCl as reference electrode. The MXene-based electrode was prepared by mixing active materials, carbon black and polyvinylidene fluoride, with a mass ratio of 8:1:1 in N-methyl pyrrolidone. The resulting slurry was coated onto copper foils and dried. All electrochemical tests were conducted using an electrochemical station (CHI660E, Chenhua, Shanghai, China) in 1 mol L^−1^ H_2_SO_4_ electrolyte at room temperature. Cycling stability was measured by repeating the galvanostatic charge–discharge test for 10,000 cycles using a battery test system (BT2018A, Lanbo, Huangshi, China).

### 3.5. Ex Situ Electrochemical XRD

XRD pattern data were collected for a series of charged and discharged samples using Rigaku Ultima IV X-ray diffractometer equipped with Cu Kα radiation (λ = 1.5418 Å). To prepare the samples in various discharged states, three-electrode cells were first activated via CV sweep at a scan rate of 10 mV s^−1^. They were then electrochemically discharged to different potentials at a scan rate of 2 mV s^−1^, before being stopped and disassembled. The electrodes were separated from the disassembled cells and washed with water. The charged samples were prepared by first discharging the cell to −0.45 V and then charging it to various voltages, such as −0.35 V, −0.25 V, −0.15 V, and −0.05 V.

### 3.6. Computational Detail

Density functional theory calculations were performed using the Vienna Ab initio Simulation Package (VASP) software 5.4.4 with the projector augmented wave (PAW) method [42,43]. Spin polarization was considered in all calculations. The convergence threshold of energy was set at 10^−5^ eV, the convergence threshold of maximum stress was set at 0.05 eV/Å, and the cut-off energy was set at 400 eV. Hubbard-U correction was applied with a U value of 4.0 eV for the 3*d* orbitals of Ti. The k-points were set to 1 × 1 × 1. The adsorption energy of H was calculated by E_b_ = E_*H_ − E_*_ − E_H_, where E_*_ represents the energy of the Ti_3_C_2_T_2_ MXene (T = O or F), E_*H_ is the total energy of MXene with the hydrogen adsorbed, and E_H_ is the energy of H.

## 4. Conclusions

Thus far, we have proposed and demonstrated that customizing the structure and surface chemistry of Ti_3_C_2_T_x_ MXene through hydrothermal treatment in NaOH solution with the reducing reagent CA is crucial for enabling high-performance supercapacitor electrodes. As revealed in Table S6, the interlayer space was expanded and homogenized by the Na^+^ intercalation and hydrothermal effect, providing enough space for ion storage. The –F terminal groups are replaced with –O terminal groups, which enhance hydrophilicity and facilitate electrolyte accessibility to the MXene’s surface. Importantly, –O groups exhibit stronger adsorption for electrolyte ions (H^+^), providing sufficient electrochemical active sites. The change in terminations further results in an increase in the valence of Ti, making it more prone to reduction. As a result, the electrochemical activity of MXene is considerably intensified, exhibiting high current densities and a pair of clear redox peaks in CV curves, as well as a twofold capacitance (543 F g^−1^ at 2 mV s^−1^) and superior cycle life compared to the unmodified sample. This work demonstrates the possibility of tuning electrochemical activity by adjusting the interlayer structure and surface chemistry of MXene, offering a facile way to enhance the capacitance of various MXenes.

## Data Availability

Not applicable.

## Supplementary Materials

The following supporting information can be downloaded at: https://www.mdpi.com/article/10.3390/molecules28155776/s1, Figure S1: XPS spectra for pristine Ti_3_C_2_T_x_ MXene and Ti_3_C_2_T_x_-NaOH. Figure S2: (a) TEM and (b) high-resolution TEM images of Ti_3_C_2_T_x_-NaOH-180 sample. Figure S3: The optimized structures and the positions of Ti atoms in Ti_3_C_2_O_2_ and Ti_3_C_2_F_2_. Figure S4: CV curves at various scan rates for (a) Ti_3_C_2_T_x_, (b) Ti_3_C_2_T_x_-NaOH, (c) Ti_3_C_2_T_x_-180. Figure S5: Equivalent circuit adopted in the simulation of EIS spectra. Figure S6: The logarithm of the current (log *i*) at a certain potential on the discharge branch of the CV curve as a function of the logarithm of the scan rate (log ν) for Ti_3_C_2_T_x_ electrode. Figure S7: The logarithm of the current (log *i*) at a certain potential on the discharge branch of the CV curve as a function of the logarithm of the scan rate (log *ν*) for Ti_3_C_2_T_x_-NaOH-180 electrode. Figure S8: CV profiles collected at (a) 2 mV s^−1^, (b) 20 mV s^−1^, (c) 50 mV s^−1^, and (d) 100 mV s^−1^ with hatched portions of the contribution of the capacitive limited processes for Ti_3_C_2_T_x_-NaOH-180 electrode. Table S1: Bader charge analysis of Ti atoms in Ti_3_C_2_O_2_. Table S2: Bader charge analysis of Ti atoms in Ti_3_C_2_F_2_. Table S3: Comparison of electrochemical performance between Ti_3_C_2_T_x_-NaOH-180 and the previously reported Ti_3_C_2_T_x_ MXene-based electrodes. Table S4: Equivalent circuit simulation results of the EIS spectra in Figure 4e. Table S5: Equivalent circuit simulation results of the EIS spectra in Figure 5f. Table S6: The main characteristics and capacitance of pristine Ti_3_C_2_T_x_ and the modified sample Ti_3_C_2_T_x_-NaOH-180. References [44,45,46,47,48,49] are cited in the supplementary materials.

## Sample Availability

Samples of the compounds are available from the authors.
