# Supplementary material for: Intensifying Electrochemical Activity of Ti3C2Tx MXene via Customized Interlayer Structure and Surface Chemistry
Source: Molecules. 2023 Jul 31;28(15):5776. doi: 10.3390/molecules28155776 (PMC10421071; doi:10.3390/molecules28155776)
Supplement: Supplementary file 1 [file molecules-28-05776-s001.zip › molecules-2513281-supplementary.pdf]

Supporting Information

Intensifying Electrochemical Activity of $Ti_3C_2T_x$ MXene via Customized Interlayer Structure and Surface Chemistry

Minmin Hu, Lihong Chen, Yunqi Jing, Yuanyuan Zhu, Jun Dai, Alan Meng, Changlong Sun, Jin Jia* and Zhenjiang Li*

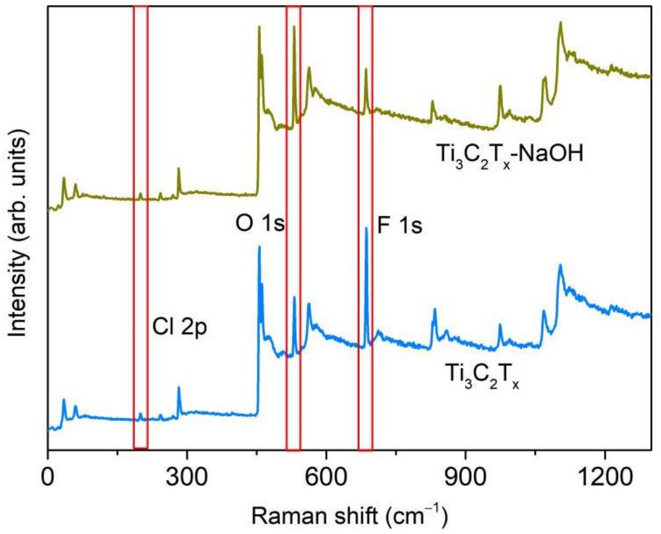

Fig. S1. XPS spectra for pristine $Ti_3C_2T_x$ MXene and $Ti_3C_2T_x$-NaOH. $Ti_3C_2T_x$ MXene prepared by etching Ti_3AlC_2 with HCl-LiF mixture were terminated with O-containing, –F and –Cl groups. The content of –Cl group in pristine MXene and the change of the –Cl group content after NaOH treatment is negligible.

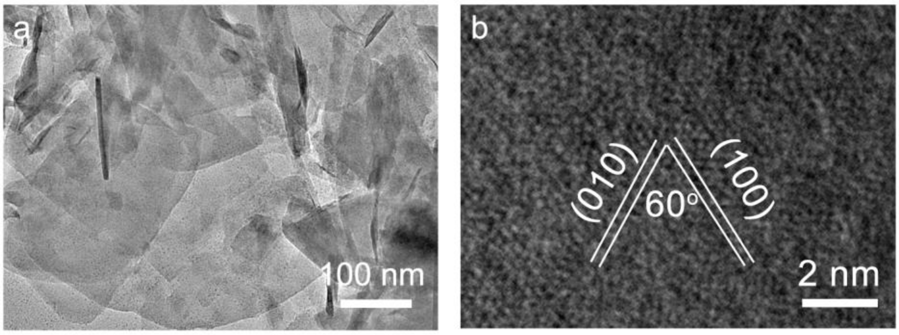

Fig. S2. (a) TEM image and (b) high-resolution TEM image of $Ti_3C_2T_x$-NaOH-180 sample. Its sheet is very thin and disperses as individual layer or stack of several layers. The lattice spacings corresponding to (100) or (010) plane is measured about 0.27 nm. (100) and (010) planes show characteristic 60°.

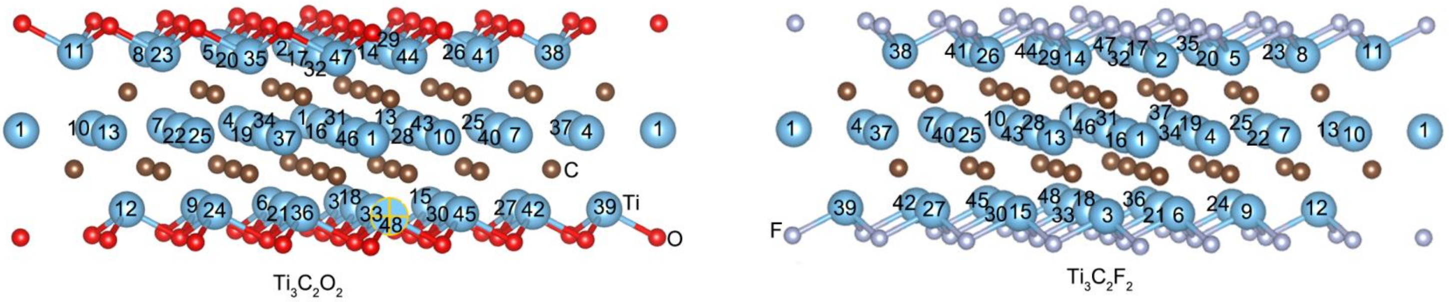

Fig. S3. The optimized structures and the positions of Ti atoms in$Ti_3C_2O_2$ and $Ti_3C_2F_2$.

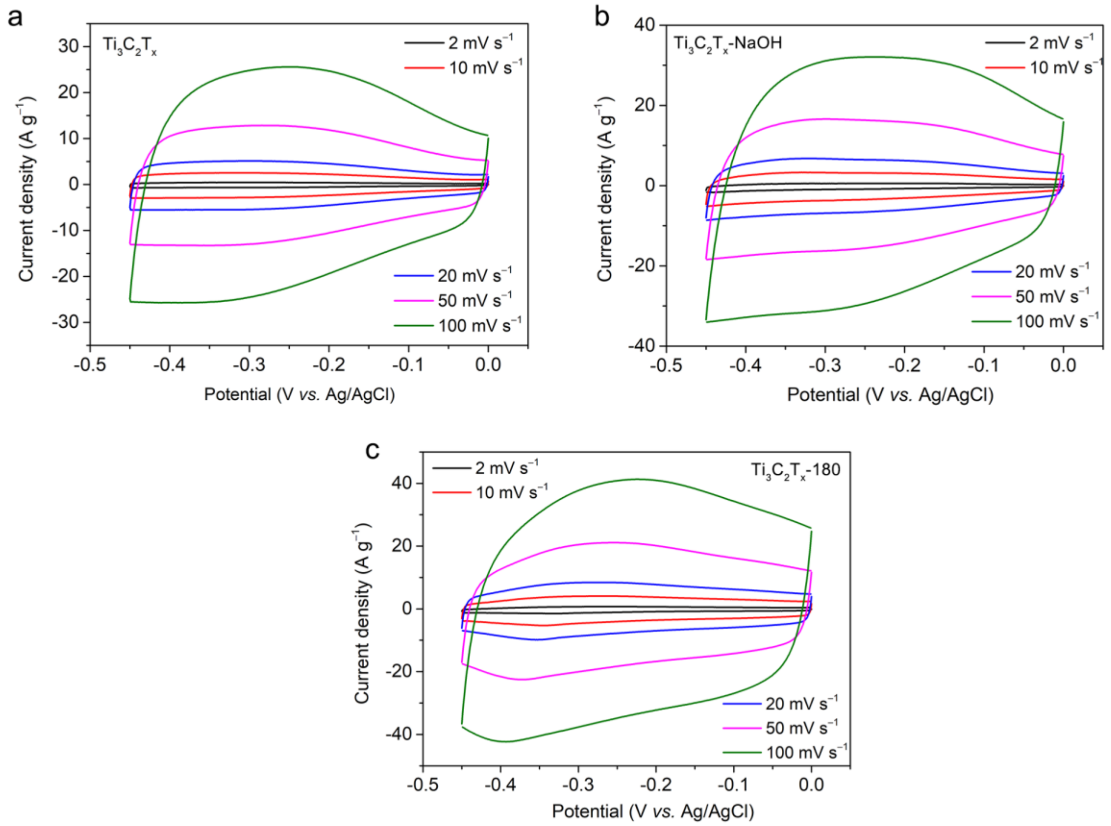

Fig. S4. CV curves at various scan rates for (a) $Ti_3C_2T_x$, (b) $Ti_3C_2T_x$-NaOH, (c) $Ti_3C_2T_x$-180.

Tab. S1. Bader charge analysis of Ti atoms in $Ti_3C_2O_2$.

Position of Ti	Charge	Position of Ti	Charge
1[a]	2.329739	25	2.314291
2	2.119117	26	2.076086
3	2.064528	27	2.071757
4	2.363035	28	2.347235
5	1.998048	29	2.923661
6	2.057172	30	2.066421
7	2.32702	31	2.283946
8	2.089243	32	2.840287
9	2.081644	33	2.028775
10	2.341395	34	2.340031
11	2.089979	35	2.080679
12	2.050423	36	2.062815
13	2.343072	37	2.344213
14	2.065652	38	2.112786
15	2.080376	39	2.090091
16	2.368623	40	2.354633
17	2.5938	41	2.055506
18	2.068876	42	2.081047
19	2.356807	43	2.370441
20	2.098028	44	2.105219
21	2.066398	45	2.063772
22	2.354323	46	2.379974
23	2.109878	47	2.106383
24	2.073624	48	2.060565

[a]number represents the positions of Ti atoms in $Ti_3C_2O_2$ as shown in Figure S3

Tab. S2. Bader charge analysis of Ti atoms in $Ti_3C_2F_2$.

Position of Ti	Charge	Position of Ti	Charge
1[a]	2.5247	25	2.456103
2	2.418699	26	2.543717
3	2.586129	27	2.467842
4	2.3742	28	2.509465
5	2.438538	29	2.357361
6	2.387828	30	2.392464
7	2.414843	31	2.459569
8	2.318103	32	2.523248
9	2.796821	33	2.448507
10	2.472359	34	2.433237
11	2.343022	35	2.460292
12	2.522362	36	2.525133
13	2.623113	37	2.460048
14	2.51442	38	2.392856
15	2.597759	39	2.565084
16	2.614421	40	2.494663
17	2.557359	41	2.394128
18	2.325047	42	2.397052
19	2.861309	43	2.729333
20	2.344036	44	2.368331
21	2.436604	45	2.387668
22	2.504105	46	2.475112
23	2.507641	47	2.491145
24	2.431864	48	2.534686

[a]number represents the positions of Ti atoms in $Ti_3C_2F_2$ as shown in Figure S3

Tab. S3. Comparison of electrochemical performance between $Ti_3C_2T_x$-NaOH-180 and the previously reported $Ti_3C_2T_x$ MXene based electrodes

**Electrode material**	**Electrolyte**	**Capacitive performance**	**Refs**
$Ti_3C_2T_x$-NaOH-180	1 mol L^{-1} H_2SO_4	543 F g^{-1} at 2 mV s^{-1}	This work
$Ti_3C_2T_x$-Ni	3 mol L^{-1} H_2SO_4	369 F g^{-1} at 2 mV s^{-1}	[1]
P–$Ti_3C_2T_x$	1 mol L^{-1} H_2SO_4	520 F g^{-1} at 2 mV s^{-1}	[2]
$Ti_3C_2T_x$–K_2SO_4–C	1 mol L^{-1} H_2SO_4	380 F g^{-1} at 2 mV s^{-1}	[3]
P–$Ti_3C_2T_x$	3 mol L^{-1} H_2SO_4	448 F g^{-1} at 1 A g^{-1}	[4]
N–$Ti_3C_2T_x$ film	1 mol L^{-1} H_2SO_4	340 F g^{-1} at 2 mV s^{-1}	[5]
N–$Ti_3C_2T_x$-300	3 mol L^{-1} H_2SO_4	415.0 F g^{-1} at 2 mV s^{-1}	[6]
N-butyllithium-treated $Ti_3C_2T_x$	1 mol L^{-1} H_2SO_4	523 F g^{-1} at 2 mV s^{-1}	[7]
N-$Ti_3C_2T_x$-200	1 mol L^{-1} H_2SO_4	192 F g^{-1} at 1 mV s^{-1}	[8]
400-KOH-$Ti_3C_2T_x$	1 mol L^{-1} H_2SO_4	517 F g^{-1} at 1 mV s^{-1}	[9]
$Ti_3C_2T_x$ hydrogel electrode	3 mol L^{-1} H_2SO_4	380 F g^{-1} at 2 mV s^{-1}	[10]
Macroporous $Ti_3C_2T_x$ electrode	3 mol L^{-1} H_2SO_4	310 F g^{-1} at 10 mV s^{-1}	[10]
d-$Ti_3C_2T_x$	1 mol L^{-1} H_2SO_4	325 F g^{-1} at 2 mV s^{-1}	[11]
$Ti_3C_2T_x$ 'clay'	1 mol L^{-1} H_2SO_4	245 F g^{-1} at 2 mV s^{-1}	[12]

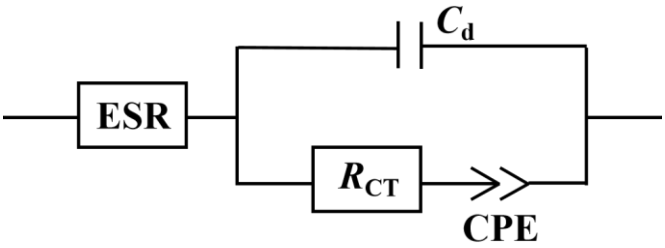

Fig. S5. Equivalent circuit adopted in the simulation of EIS spectra. ESR: equivalent series resistance ; C_d: electrical double layer capacitor; R_{ct}: charge transfer resistance; CPE: constant phase angle element.

Tab. S4. Simulation results of the EIS spectra (Figure 4e) of the prepared electrodes at open-circuit potentials (~0 V) *vs.* Ag/AgCl

Elements	*ESR*/ohm	C_d/F	R_{ct}/ohm	CPE-α	CPE-*P*
Pristine $Ti_3C_2T_x$	0.8845	0.0002913	0.2519	0.08075	0.8373
$Ti_3C_2T_x$-NaOH	0.8483	0.000456	0.1414	0.1194	0.8392
$Ti_3C_2T_x$-180	0.6921	0.0004942	0.1628	0.1465	0.8359
$Ti_3C_2T_x$-NaOH-180	0.6888	0.000441	0.1426	0.2712	0.7293

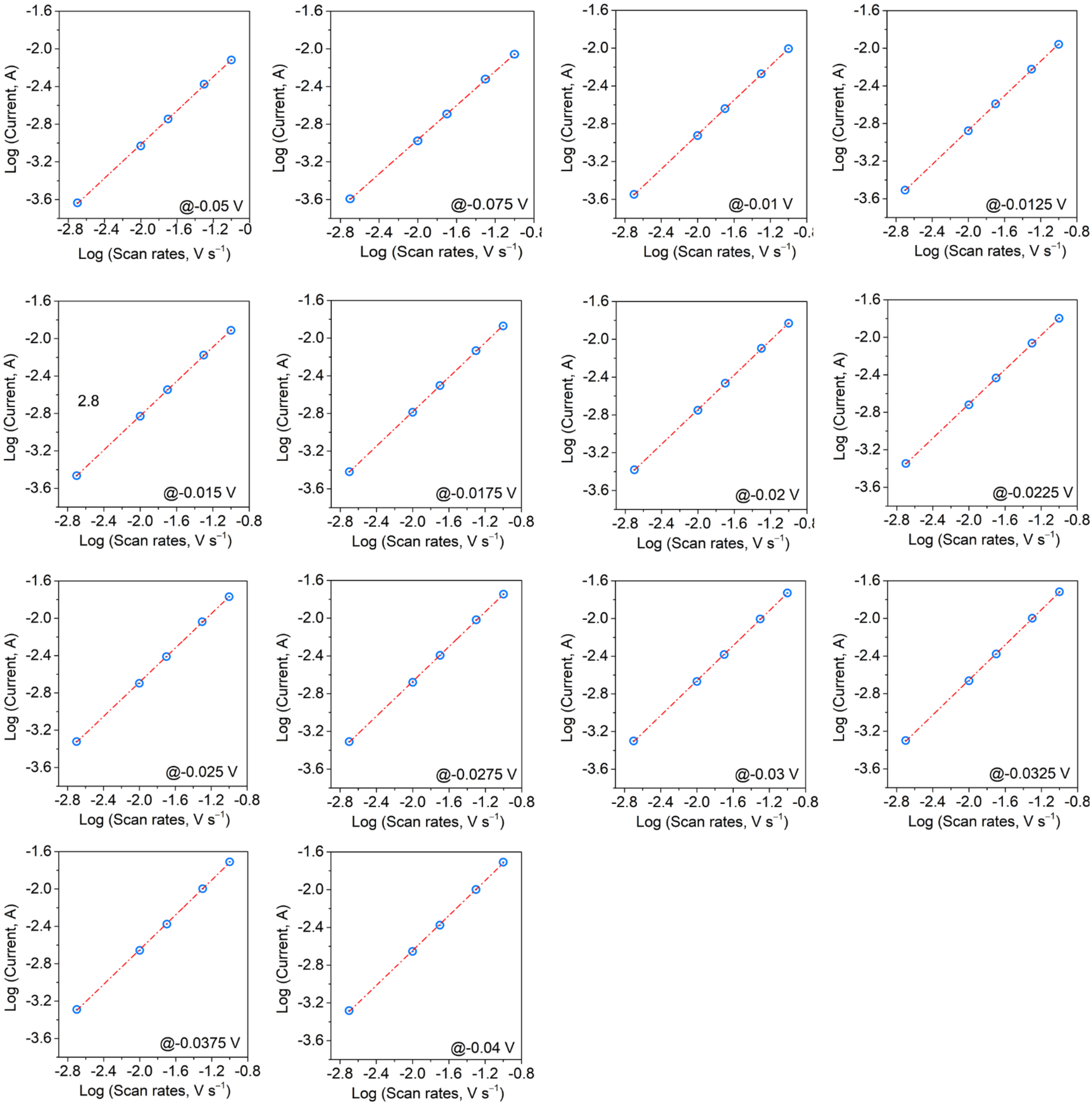

Fig. S6. The logarithm of the current (log *i*) at a certain potential on the discharge branch of the CV curve as a function of the logarithm of the scan rate (log ν) for $Ti_3C_2T_x$ electrode. The *b* value was obtained by estimating the slope of the curve.

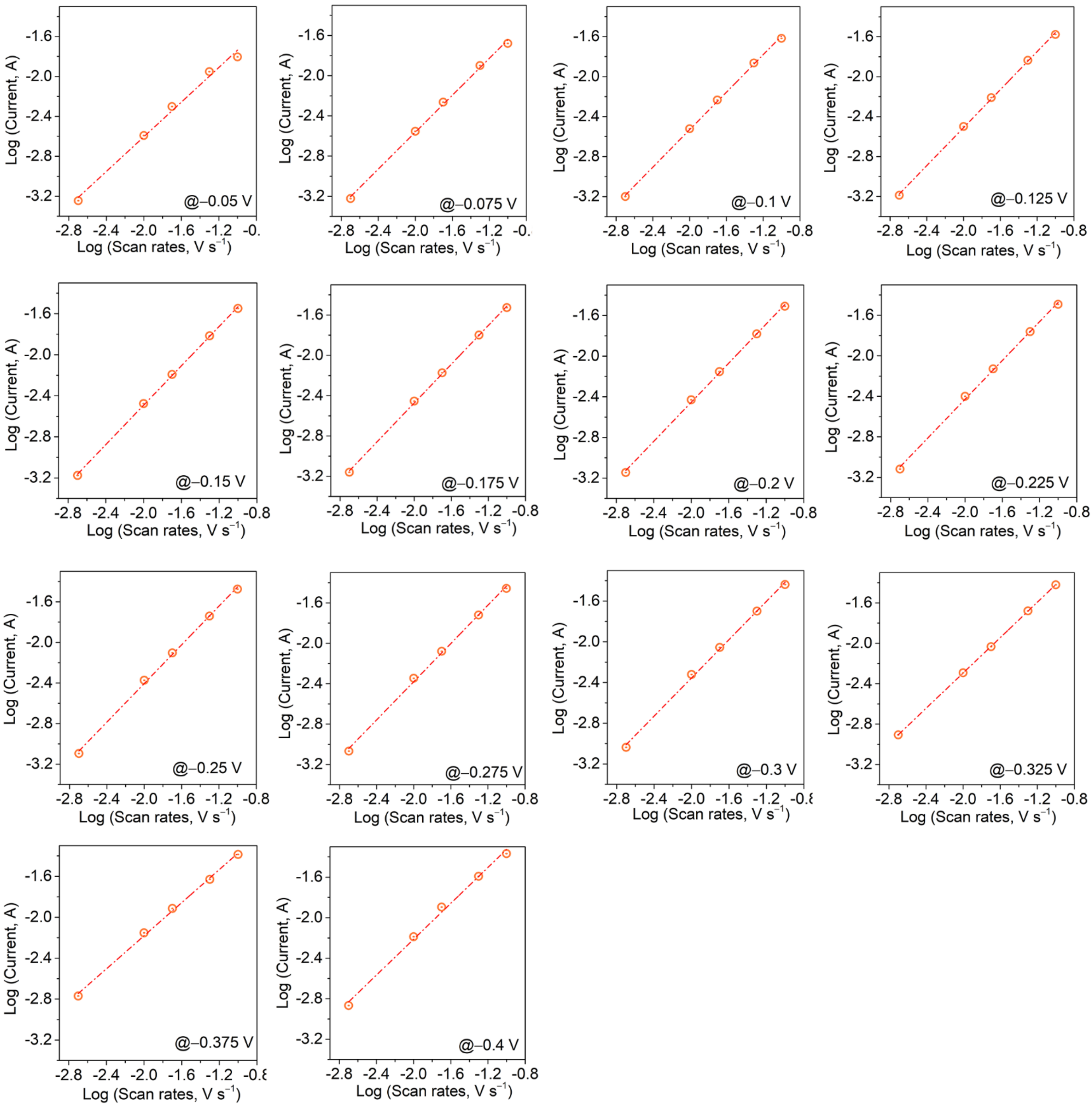

Fig. S7. The logarithm of the current (log *i*) at a certain potential on the discharge branch of the CV curve as a function of the logarithm of the scan rate (log *v*) for $Ti_3C_2T_x$-NaOH-180 electrode. The *b* value was obtained by estimating the slope of the curve.

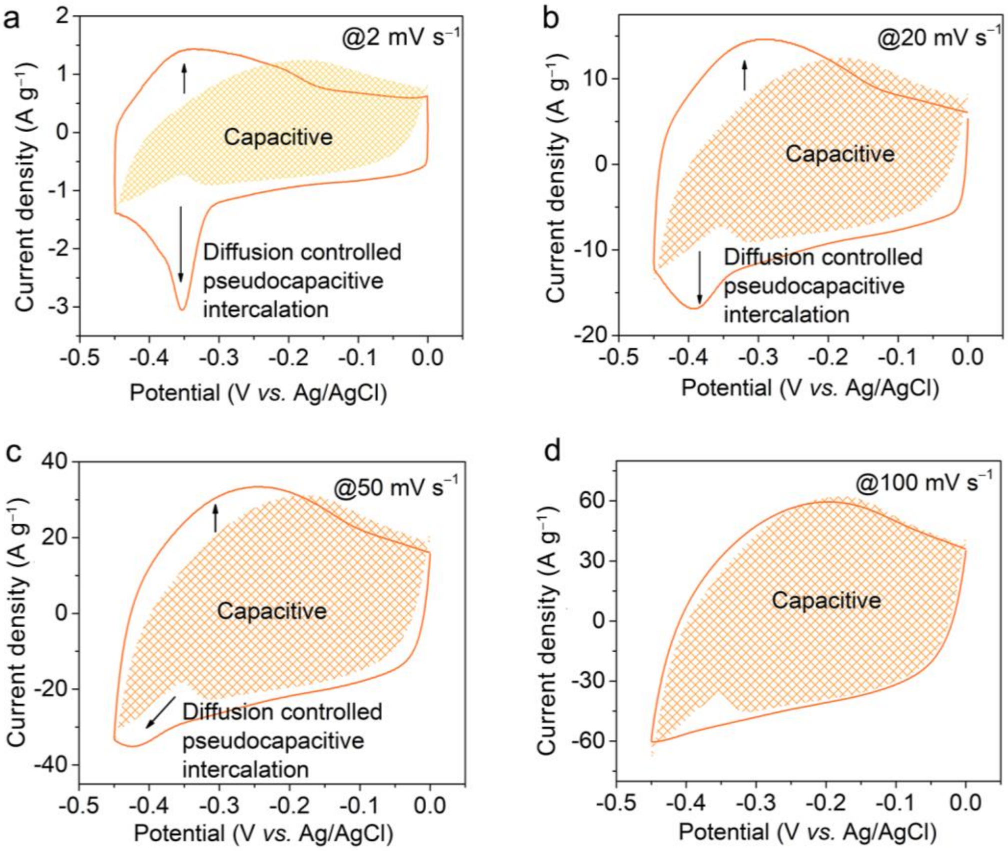

Fig. S8. CV profiles collected at (a) 2 mV s^{-1}, (b) 20 mV s^{-1}, (c) 50 mV s^{-1}, (d) 100 mV s^{-1} with hatched portions of the contribution of the capacitive limited processes for $Ti_3C_2T_x$-NaOH-180 electrode. There is a noticeable and prevailing contribution of diffusion-limited processes to the total capacitance.

Tab. S5. Simulation results of the EIS spectra of of the $Ti_3C_2T_x$-NaOH-180 electrode collected at different potentials versus Ag/AgCl (Figure 5f)

Elements	R_s/ohm	C_d/F	R_{ct}/ohm	CPE-P	CPE-α
–0.12 V	0.8268	0.0002054	0.3943	0.204	0.8618
–0.20 V	0.8351	0.0002485	0.3725	0.2162	0.8614
–0.27 V	0.8485	0.000469	0.3561	0.2311	0.8698
–0.34 V	0.9721	0.02024	0.4273	0.2838	0.856
–0.4 V	0.8247	0.0004174	0.3252	0.3002	0.7621

Tab. S6. The main characteristics and capacitance of pristine $Ti_3C_2T_x$ and the modified sample-$Ti_3C_2T_x$-NaOH-180

Sample	c-lattice parameter	FWHM of (002) peak	Content of O-containing groups	Content of Ti^{3+} or Ti^{4+}	Contact angle of water	Capacitance at 2 mV s^{-1}
$Ti_3C_2T_x$	22.03 Å	0.634	Low	Low	56°	217 F g^{-1}
$Ti_3C_2T_x$-NaOH-180	24.00 Å	0.375	High	High	12°	543 F g^{-1}

References

[1] Xu, H.; Fan, J.; Su, H.; Liu, C.; Chen, G.; Dall' Agnese, Y.; Gao, Y. Metal ion-induced porous MXene for all-solid-state flexible supercapacitors. *Nano Lett.* **2023**, *23*, 283−290.

[2] Chen, L.; Bi, Y.; Jing, Y.; Dai, J.; Li, Z.; Sun, C.; Meng, A.; Xie, H.; Hu, M. Phosphorus doping strategy-induced synergistic modification of interlayer structure and chemical state in $Ti_3C_2T_x$ toward enhancing capacitance. *Molecules* **2023**, *28*, 4892.

[3] Li, Z.; Dai, J.; Li, Y.; Sun, C.; Meng, A.; Cheng, R.; Zhao, J.; Hu, M.; Wang, X. Intercalation-deintercalation design in MXenes for high performance supercapacitors. *Nano Res.* **2022**, *15*, 3213−3221.

[4] Gupta, N.; Sahu, R. K.; Mishra, T.; Bhattacharya, P. Microwave-assisted rapid synthesis of titanium phosphate free phosphorus doped Ti_3C_2 MXene with boosted pseudocapacitance. *J. Mater. Chem. A* **2022**, *10*, 15794–15810.

[5] Zhang, T.; Xiao, J.; Li, L.; Zhao, J.; Gao, H. A high-performance supercapacitor electrode based on freestanding N-doped $Ti_3C_2T_x$ film. *Ceram. Int.* **2020**, *46*, 21482–21488.

[6] Tian, Y.; Que, W.; Luo, Y.; Yang, C.; Yin, X.; Kong, L. B. Surface nitrogen-modified 2D titanium carbide (MXene) with high energy density for aqueous supercapacitor applications. *J. Mater. Chem. A* **2019**, *7*, 5416–5425.

[7] Chen, X.; Zhu, Y.; Zhang, M.; Sui, J.; Peng, W.; Li, Y.; Zhang, G.; Zhang, F.; Fan, X. N-butyllithium-treated $Ti_3C_2T_x$ MXene with excellent pseudocapacitor performance. *ACS Nano* **2019**, *13*, 9449−9456.

[8] Wen, Y.; Rufford, T. E.; Chen, X.; Li, N.; Lyu, M.; Dai L.; Wang, L. Nitrogen-doped $Ti_3C_2T_x$ MXene electrodes for high-performance supercapacitors. *Nano Energy* **2017**, *38*, 368–376.

[9] Li, J.; Yuan, X.; Lin, C.; Yang, Y.; Xu, L.; Du, X.; Xie, J.; Lin J.; Sun, J. Achieving high pseudocapacitance of 2D titanium carbide (MXene) by cation intercalation and surface modification. *Adv. Energy Mater.* **2017**, *7*, 1602725.

[10] Lukatskaya, M. R.; Kota, S.; Lin, Z.; Zhao, M.; Shpigel, N.; Levi, M. D.; Halim, J.; Taberna, P. -L.; Barsoum, M. W.; Simon, P.; Gogotsi, Y. Ultra-high-rate pseudocapacitive energy storage in two-dimensional transition metal carbides. *Nat. Energy* **2017**, *2*, 17105.

[11] Dall' Agnese, Y.; Lukatskaya, M. R.; Cook, K. M.; Taberna, P. -L.; Gogotsi, Y.; Simon, P. High capacitance of surface-modified 2D titanium carbide in acidic electrolyte. *Electrochem. Commun.* **2014**, *48*, 118–122.

[12] Ghidiu, M.; Lukatskaya, M. R.; Zhao, M. Q.; Gogotsi, Y.; Barsoum, M. W. Conductive two-dimensional titanium carbide 'clay' with high volumetric capacitance. *Nature* **2014**, *516*,78–81.
